# Supplementary material for: Origins of Barents-Kara sea-ice interannual variability modulated by the Atlantic pathway of El Niño–Southern Oscillation
Source: Nat Commun. 2023 Feb 3;14:585. doi: 10.1038/s41467-023-36136-5 (PMC9898563; doi:10.1038/s41467-023-36136-5)
Supplement: Supplementary file 1 — Supplementary Information [file 41467_2023_36136_MOESM1_ESM.pdf]

Supplementary information file of "Origins of Barents-Kara sea-ice interannual variability modulated by the Atlantic pathway of El Niño–Southern Oscillation"

Binhe Luo[1)], Dehai Luo[2)], Yao Ge[2)], Aiguo Dai[3)], Lin Wang[4)], Ian Simmonds[5)], Cunde Xiao[1)], Lixin Wu[6)], and Yao Yao[2)]

(1) State Key Laboratory of Earth Surface Processes and Resource Ecology, Beijing Normal University, Beijing 100032, China

(2) Key Laboratory of Regional Climate-Environment for Temperate East Asia, Institute of Atmospheric Physics, Chinese Academy of Sciences and University of Chinese Academy of Sciences, Beijing 100029, China

(3) Department of Atmospheric and Environmental Sciences, University at Albany, State University of New York, Albany, NY 12222, USA

(4) Center for Monsoon System Research, Institute of Atmospheric Physics, Chinese Academy of Sciences, Beijing 100029, China

(5) School of Geography, Earth and Atmospheric Sciences, University of Melbourne, Parkville, Victoria 3010, Australia

(6) Frontiers Science Center for Deep Ocean Multispheres and Earth System and Key Laboratory of Physical Oceanography, Ocean University of China, Qingdao 266100, China and Laoshan Laboratory, Qingdao 266237, China

Corresponding authors: Dr. Dehai Luo, Institute of Atmospheric Physics, Chinese Academy of Sciences, Beijing, email: ldh@mail.iap.ac.cn and Dr. Cunde Xiao, State Key Laboratory of Earth Surface Processes and Resource Ecology, Beijing Normal University, Beijing, China, email: cdxiao@bnu.edu.cn

Supplementary Table 1. Descriptions of 34 Coupled Model Intercomparison Project phase 6 (CMIP6) models. Names of 34 CMIP6 models used in this paper

Model	Institution
ACCESS-CM2	CSIRO-ARCCSS-BoM
ACCESS-ESM1-5	CSIRO
BCC-CSM2-MR	BCC
BCC-ESM1	BCC
CAMS-CSM1-0	CAMS
CanESM5	CCCma
CAS-ESM2-0	CAS
CESM2	NCAR
CESM2-FV2	NCAR
CESM2-WACCM	NCAR
CESM2-WACCM-FV2	NCAR
CMCC-CM2-SR5	CMCC
CMCC-ESM2	CNRM-CERFACS
EC-Earth3	EC-Earth-Consortium
EC-Earth3-AerChem	EC-Earth-Consortium
EC-Earth3-CC	EC-Earth-Consortium
EC-Earth3-Veg	EC-Earth-Consortium
EC-Earth3-Veg-LR	EC-Earth-Consortium
FGOALS-f3-L	CAS
FGOALS-g3	CAS
FIO-ESM-2-0	FIO-QLNM
IPSL-CM6A-LR	IPSL
PSL-CM5A2-INCA	PSL
MIROC6	MIROC
MPI-ESM-1-2-HAM	MRI
MPI-ESM1-2-HR	MRI
MPI-ESM1-2-LR	MRI
MRI-ESM2-0	MRI
NESM3	NUIST
NorCPM1	NCC
NorESM2-LM	NCC
NorESM2-MM	NCC
SAM0-UNICON	SNU
TaiESM1	AS-RCEC

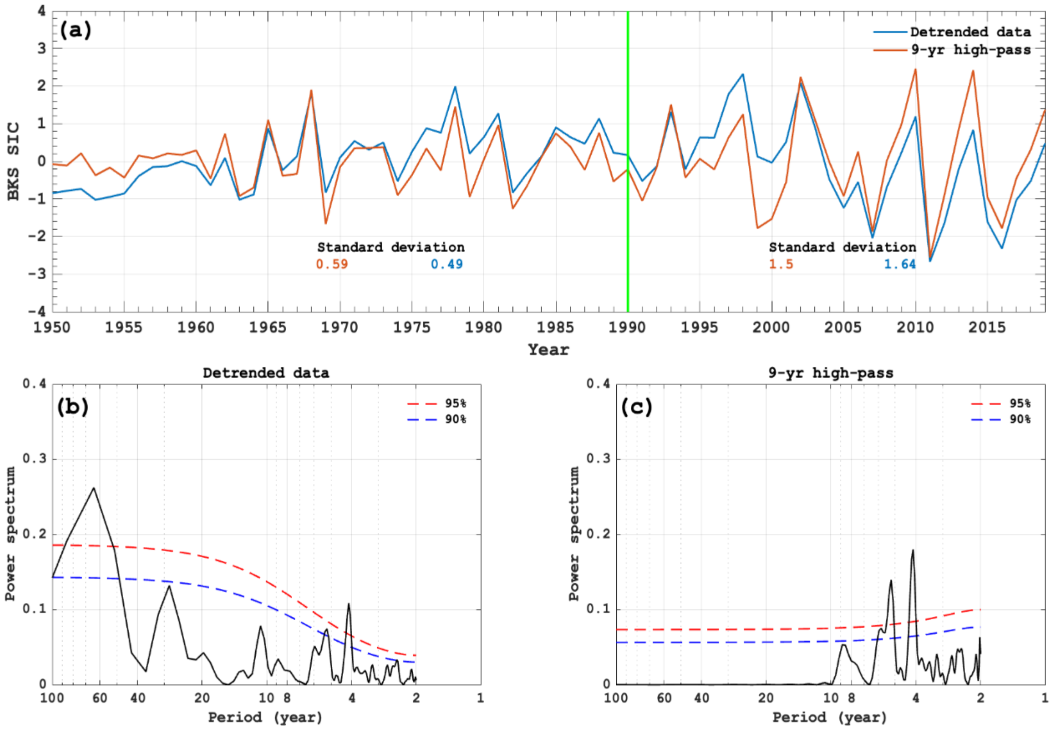

Supplementary Figure 1. Interannual variations and power spectra of the winter Barents-Kara sea-ice for the ERA data. (a) Time series of normalized DJF (December-February) mean Arctic sea ice concentration (SIC) anomaly averaged over Barents-Kara Seas (BKS) (30°-90°E, 65°-85°N) for detrended (blue line) and 9-yr high-pass filtered (black line) ERA5 data, where the blue (red) value in the left- (right-) hand side of the green line represents the mean standard deviations of the BKS SIC variations averaged over 1950-1990 (1991-2019). **(b, c)** Power Spectra of the (b) detrended and (c) 9-yr high-pass filtered BKS SIC time series. In panels b-c, the blue (red) dashed line represents the 90% (95%) confidence level.

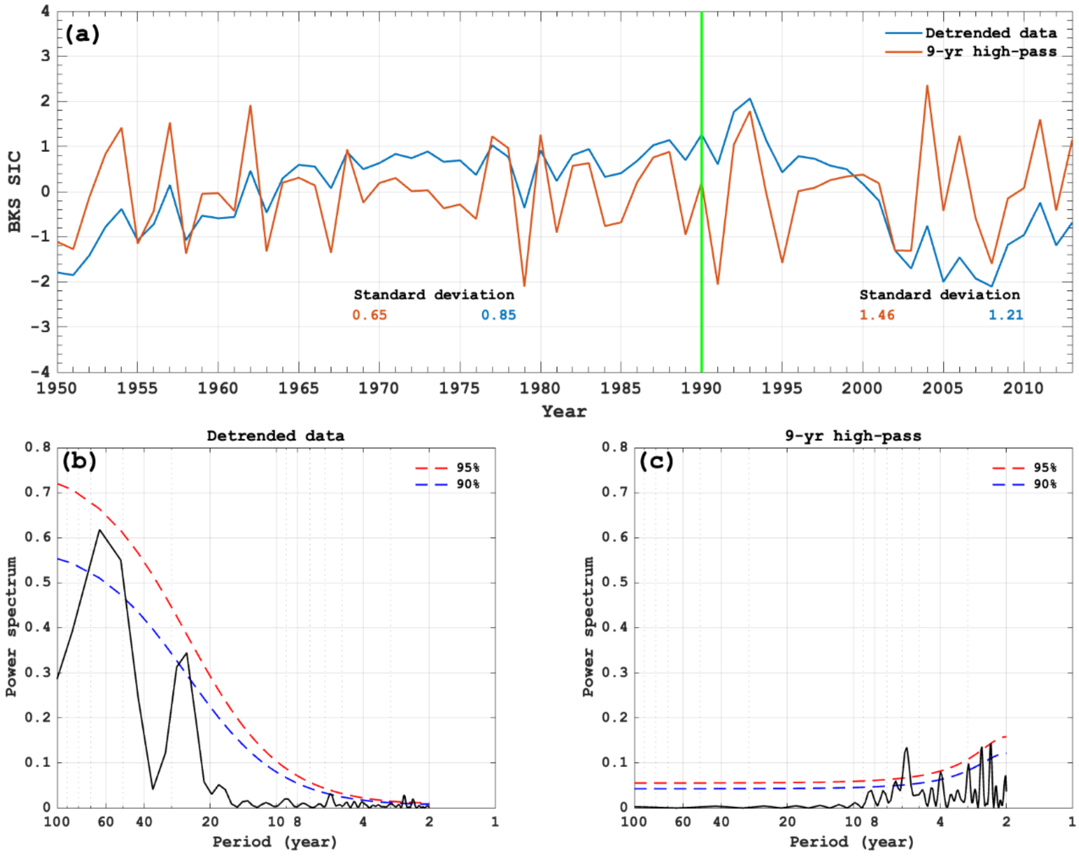

Supplementary Figure 2. Interannual variations and power spectra of the winter Barents-Kara sea-ice for the ensemble results of 34 CMIP6 models. Same as Supplementary Figure 1 but for 34 CMIP6 models shown in Supplementary Table 1. The correlation coefficient of the CMIP6 ensemble DJF (December-February) mean sea ice concentration (SIC) over Barents-Kara Seas (BKS) with the Niño3.4 index is 0.11 during 1950-1989 and -0.08 during 1990-2013.

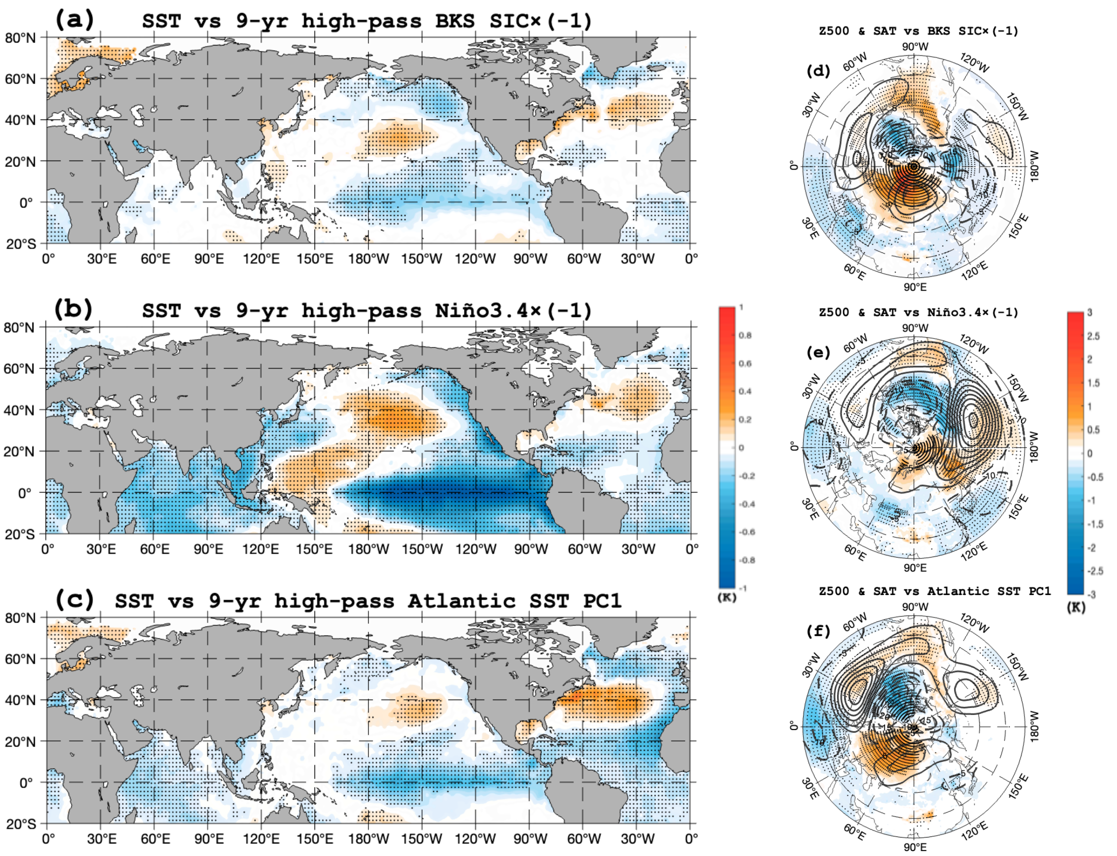

Supplementary Figure 3. Spatial patterns of winter sea surface temperature (SST), 500-hPa geopotential height (Z500) and surface air temperature (SAT) anomalies related to the interannual variations of the sea ice concentration (SIC) over Barents-Kara Seas (BKS), North Atlantic SST and Niño3.4 index during1950-2021. (a, b, c) DJF (December-February) mean sea surface temperature (SST), (d, e, f) 500-hPa geopotential height (Z500, contour interval=10, gpm) and surface air temperature (SAT, color shading, K) anomalies regressed onto 9-yr high-pass normalized **(a, d)** BKS SIC, (**b, e**) Niño3.4 and (**c, f**) Atlantic SST PC1 time series during1950-2021. The dot represents the region being significant at the 95% confidence level based on a two-sided student t-test.

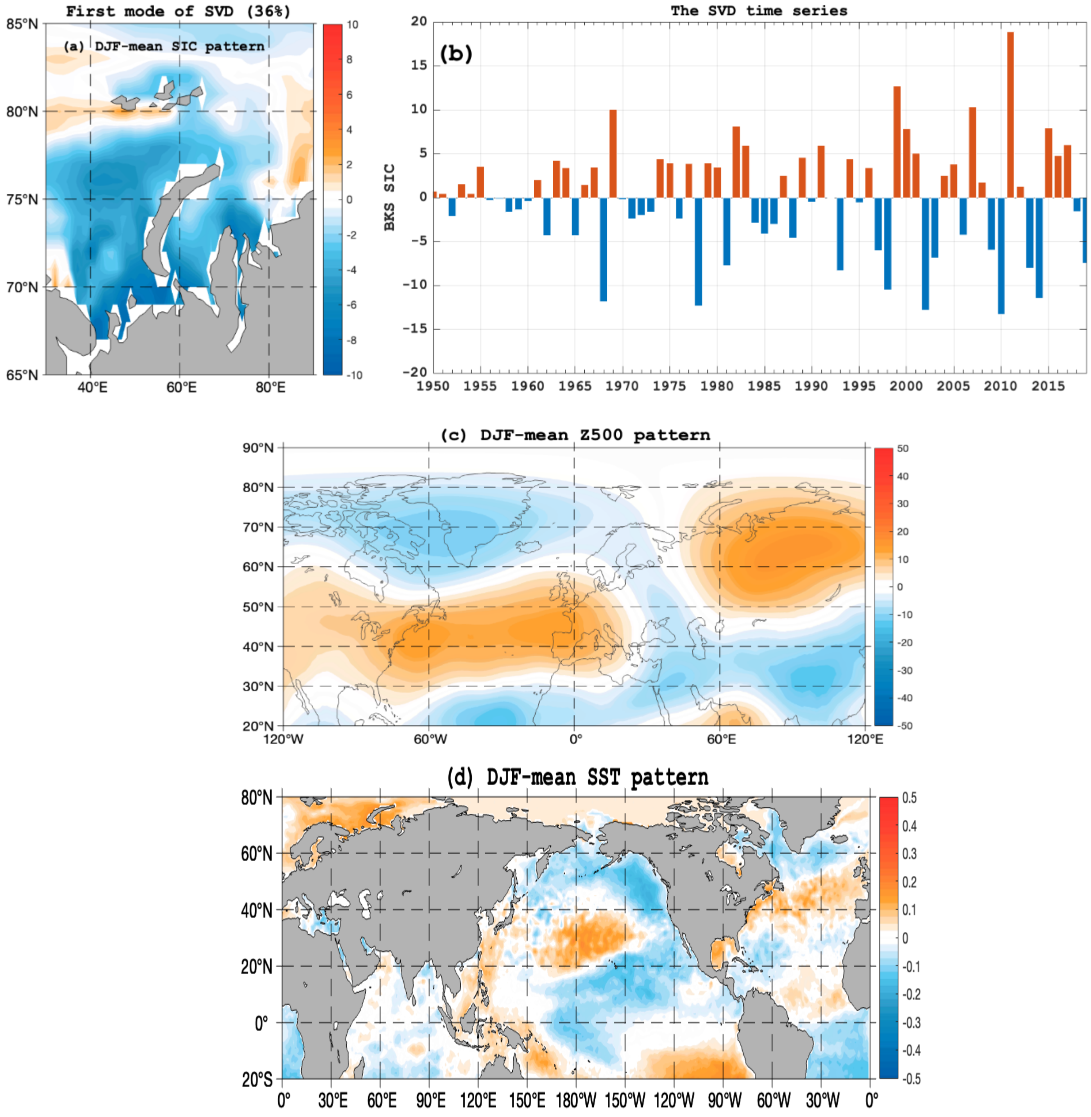

Supplementary Figure 4. The first singular value decomposition (SVD) mode of the winter sea ice concentration (SIC) anomaly (color shading, %) over Barents-Kara Seas (BKS, 30°-90°E, 65°-85°N) and its co-variability fields with winter 500-hPa geopotential height (Z500, color shading, gpm) and sea surface temperature (SST, K) anomalies. **(a)** Spatial pattern of the SVD mode of the DJF (December-February) mean SIC anomaly (color shading, %) over BKS associated with DJF-mean Z500 (color shading, gpm) anomaly and (**b**) the SVD time series of the BKS SIC during 1950-2019. **(c, d)** Dominant patterns of the co-variability of DJF-mean (c) Z500 (color shading, gpm) and (d) SST (color shading, K) anomalies with the BKS SIC as obtained from the Maximum covariance analysis (or SVD).

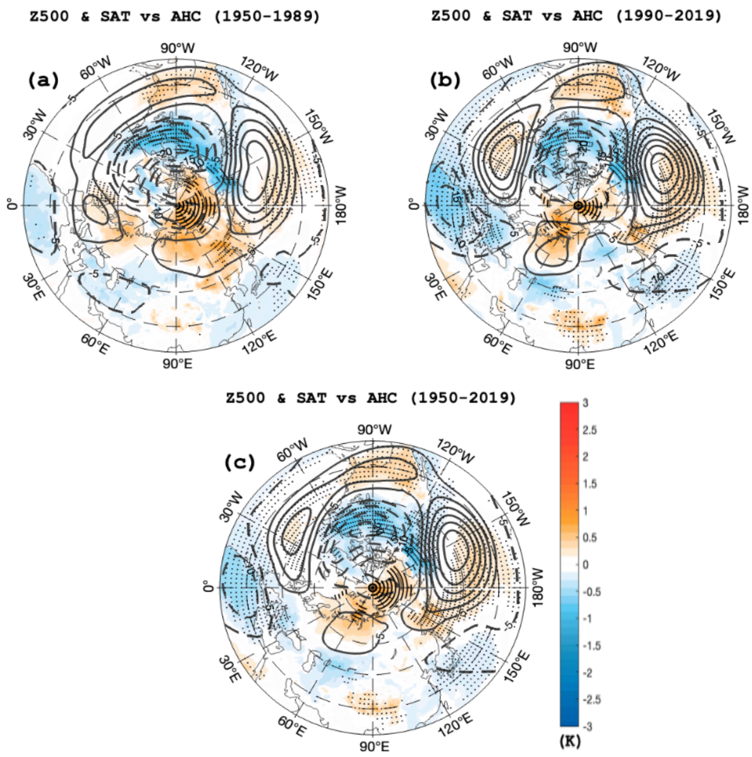

Supplementary Figure 5. Responses of winter atmospheric fields to the interannual variability of the Atlantic Hadley cell. (**a, b, c**) Regressed DJF (December-February) mean 500-hPa geopotential height (Z500, contour interval=5, gpm) and surface air temperature (SAT, color shading, K) anomalies onto the time series of normalized DJF-mean Atlantic Hadley cell index during (**a**) 1950-1989, **(b)** 1990-2019 and **(c)** 1950-2019, where the dot represents the region being the 95% confidence level based on a two-sided student t-test.
